# Optimizing Intermittent Hypoxic–Hyperoxic Training for Safety and Feasibility: An Exploratory Pilot Study

**DOI:** 10.3390/jfmk11030258

**Published:** 2026-06-29

**Authors:** Manuel Marzola, Tommaso Antonio Giacon, Simona Mrakic-Sposta, Costantino Balestra, Alessandra Vezzoli, Stefano Zappalà, Simona Stimolo, Michele Lazzari, Katia Battista, Margherita Bortolato, Giulia D’Amico, Gerardo Bosco

**Affiliations:** 1 Department of Medicine and Aging Sciences, University “G. d’Annunzio” Chieti-Pescara, 66100 Chieti, Italy; manuel.marzola@phd.unich.it (M.M.); gerardo.bosco@unich.it (G.B.); 2 Department of Biomedical Sciences, University of Padova, 35122 Padova, Italy; stefano.zappala.med@gmail.com (S.Z.); simonastimolo1996@gmail.com (S.S.); michelelazzari8@gmail.com (M.L.); katiabattista99@gmail.com (K.B.); margherita.bortolato00@gmail.com (M.B.); giulia.damico89@gmail.com (G.D.); 3 Institute of Clinical Physiology, National Research Council (IFC-CNR), Piazza dell’Ospedale Maggiore 3, 20162 Milan, Italy; alessandra.vezzoli@cnr.it; 4 Environmental, Occupational, Ageing (Integrative) Physiology Laboratory, Haute Ecole Bruxelles-Brabant (HE2B), 1180 Brussels, Belgium; costantino.balestra@ulb.be

**Keywords:** intermittent hypoxic–hyperoxic training, systemic inflammation, metabolic health, low-intensity exercise

## Abstract

**Background:** Intermittent Hypoxic–Hyperoxic Training (IHHT) induces physiological adaptations. While its efficacy in athletic performance remains debated, IHHT improves health markers in pathological and geriatric populations. This Exploratory Pilot Study aimed to explore the safety and feasibility of two IHHT protocols through preliminary responses. **Methods:** Twelve healthy volunteers completed a 4-week intervention (two sessions/week, 45 min/session) combining IHHT simultaneously during low-intensity exercise. The study compared a Training Group (TG: 30 min hypoxia, 7.5 min normoxia, 7.5 min hyperoxia) with a Conditioning Group (CG: 15 min hypoxia, 22.5 min normoxia, 7.5 min hyperoxia). Outcomes assessed included cardiorespiratory parameters, Acute Mountain Sickness symptoms, Perceived Exertion, a comprehensive biochemical panel, systemic inflammation, oxidative stress, and renal status. **Results:** Both protocols were well-tolerated. The TG exhibited significantly greater oxygen desaturation than the CG (*p* = 0.048). Moreover, the CG demonstrated a significantly attenuated increase in Interleukin-6 (*p* = 0.021) compared to the TG. Additionally, preliminary variations highlighted an interesting reduction in lipid parameters (TC, LDL, and Apo A1/B ratios) in both groups, although these did not reach statistical significance after FDR correction. **Conclusions:** While both protocols proved feasible and safe, a more balanced hyperoxic-to-hypoxic exposure (CG) showed better acute physiological tolerability, attenuating cardiovascular strain and mitigating systemic pro-inflammatory responses compared to the unbalanced exposure (TG). Finally, the preliminary variations observed in lipid parameters provide a rationale that warrants further controlled investigations.

## 1. Introduction

Low-to-moderate-altitude training has traditionally been used to improve sea-level performance owing to hematological and non-hematological adaptations [1]. The physiological rationale resides in the hypobaric environment at high altitude, where the reduction in the partial pressure of oxygen decreases systemic availability. As a result, oxygen-sensing mechanisms detect oxygen deficiency and stimulate the hypoxia-inducible factor 1α (HIF-1α) to produce erythropoiesis [2], angiogenesis [3], and metabolic regulation [4].

The widespread availability of devices capable of inducing systemic or local hypoxia has facilitated the development of the ‘Live Low–Train High’ (LLTH) protocol, consisting of short hypoxic training, approximately two to five sessions per week (<3 h) [5]. This approach has proven to be effective for improving athletic performance [4], lower hypertension, and as a non-invasive and non-pharmacological therapeutic approach for specific pathologies [6,7]. Simultaneously, it is able to minimize the side effects typically associated with high-altitude exposure, such as Acute Mountain Sickness (AMS) [8].

In recent years, LLTH has been combined with normoxic or hyperoxic periods, leading to new training strategies like Intermittent Hypoxic Exposure (IHE) or Intermittent Hypoxic–Hyperoxic Exposure (IHHT). Although the IHE effect as a performance boost is still debated [9,10], evidence regarding its efficacy as a non-invasive pharmacological adjunctive therapy in various chronic cardiovascular and metabolic conditions is encouraging [11,12,13,14,15]. Similarly, IHHT has demonstrated efficacy in improving exercise capacity and cognitive function in pathological and geriatric patients [16,17,18,19].

Interestingly, similarly to hypoxic exposure, transient hyperoxia can elicit HIF-1α and ROS production in a time- and dose-dependent manner [20,21,22]. This is known as the Normobaric Oxygen Paradox (NOP), in which a rapid shift from pure normobaric oxygen to normobaric normoxic air is detected by cellular sensors as if the body were in a hypoxic state [23,24]. Transient ROS production during physical exercise or hyperoxic exposure has been shown to act as a fundamental molecular messenger rather than a damaging factor; in fact, when human cells detect an increase in ROS, they trigger the transcription factor Nrf2, which upregulates the synthesis of antioxidant proteins [20,25]. This time-dependent increase in ROS also modulates the immune system; specifically, it induces macrophage polarization toward the M2 phenotype, known for its anti-inflammatory role [20].

Moreover, a low-intensity workout performed under a modified oxygen supply is hypothesized to induce a metabolic load and ROS stimulus similar to higher-intensity exercise [23], and at the same time, hyperoxia can be used to attenuate hypoxic stress during exercise, to increase the maximal power output, and to reduce the recovery time which are the main limits of pure hypoxic training [26,27].

However, the hypoxia–hyperoxia response is complex; therefore, studies investigating the acute and chronic dose–response relationship in IHHT are necessary to maximize physiological adaptations and reduce potential side effects [7]. At the moment, IHHT has demonstrated safety and significant clinical results in pathological or geriatric populations when applied sequentially. The effects of simultaneous application remain unknown, and no studies have been conducted. On the other hand, the optimal hypoxic–hyperoxic dose aligns with the principle of hormesis, in which moderate stimuli can trigger adaptive protective responses, whereas excessive doses can induce maladaptations [28].

This Exploratory Pilot Study aimed to assess the feasibility and safety of combining hypoxic–hyperoxic exposure during low-intensity exercise. In order to find the best dose–response, two Intermittent Hypoxic–Hyperoxic Training (IHHT) protocols with different hypoxic–hyperoxic ratios have been selected to provide varying degrees of intensity. The investigation of healthy subjects represents the initial phase in the eventual extension of this to specific populations. Moreover, it could serve as a future strategy for primary prevention in healthy individuals, hypothetically inducing a hormetic response that might accelerate the benefits of exercise.

## 2. Materials and Methods

### 2.1. Study Design

This non-randomized longitudinal interventional pilot study involved 12 young and healthy volunteers who were recruited for four consecutive weeks, engaging them twice a week for a total of eight workouts.

This early-stage study was designed to assess the feasibility, safety, and acute physiological tolerability of two different IHHT protocols during exercise. To continuously monitor safety and quantify physiological tolerability to the hypoxic stimulus, SpO_2_ was designated a priori as the primary physiological outcome; HR, perception of effort, and symptoms (AMS) were established as secondary safety and tolerability endpoints. Finally, comprehensive systemic responses, including lipid, oxidative, and renal profiles, as well as inflammatory cytokines, were defined as secondary exploratory outcomes to identify potential metabolic trends for future research. For these reasons, a formal power calculation was not applicable. Instead, a total sample size of 12 participants was selected due to the novelty of the combination of hypoxic–hyperoxic intervention during exercise; this number was considered adequate to evaluate protocol feasibility and safety, while minimizing unnecessary participant exposure to an experimental paradigm, in accordance with standard pilot study methodologies [29]. Participants were assigned to the training protocols using convenience sampling based on recruitment order and participant availability; formal randomization and blinding procedures were not performed. A normoxic control group was not included to remain strictly focused on the comparison of two IHHT doses.

During the training sessions, subjects were asked to perform low standardized physical exercise: treadmill walking at 3.5/4.5 km/h, with 60 to 70 steps/min. The energy intensity was set to about four metabolic equivalents (METs). The hypoxic and hyperoxic conditions were set via a prototype machine, OX2 Master (Z-Health s.r.l., Noventa, Vicenza, Italy).

A physician supervised the sessions, and the subjects were instructed to discontinue the protocol upon the onset of intolerable symptoms. Heart rate (HR) and peripheral oxygen saturation (SpO_2_) were monitored with a pulse oximeter (GIMA Oxy 50, GIMA S.p.A., Milan, Italy). Moreover, HR was monitored with a chest band (Garmin HMR pro; Garmin Descent MKII, Garmin, Kansas City, KS, USA). Safety measures included the possibility to administer high flow 100% oxygen to the subjects and to manually ventilate them in case of emergency.

The subjects were allocated to either the Training Group (TG) or the Conditioning Group (CG) and were exposed to hypoxia (FiO_2_ 14 ± 1%), normoxia (FiO_2_ 21 ± 1%), and hyperoxia (FiO_2_ 30 ± 1%).

The TG protocol consisted of 5 min of hypoxia, followed by 2.5 min of normoxia, 5 min of hypoxia, and 2.5 min of hyperoxia. This sequence was repeated three times for a total duration of 45 min, resulting in 30 min under hypoxic conditions and 7.5 min under hyperoxic conditions.

**Figure 1:**
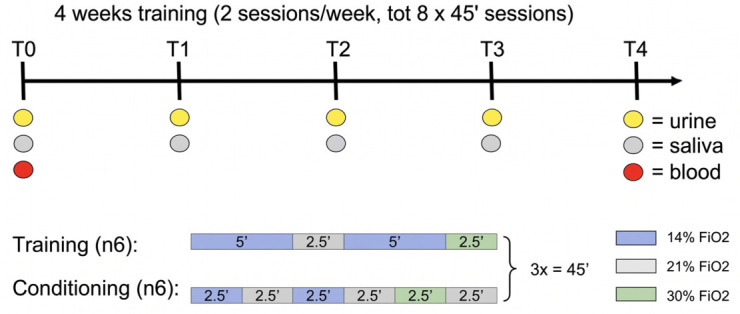
Schematic representation of the 4-week IHHT intervention. Timeline of sample collection: T0, baseline; T1, after two training sessions; T2, after four training sessions; T3, after six training sessions; T4, final, after eight training sessions. The two training and conditioning protocols differ in the duration of the hypoxia period.

The CG protocol consisted of 2.5 min of hypoxia followed by 2.5 min of normoxia, repeated twice, and 2.5 min of hyperoxia followed by 2.5 min of normoxia, repeated three times. The total duration was 45 min, resulting in 15 min under hypoxic conditions and 7.5 min under hyperoxic conditions. Details of the protocols are reported in Figure 1. The main difference was therefore in the duration of the hypoxic period.

The specific time intervals for the TG and CG protocols were designed to compare two distinct intermittent hypoxic–hyperoxic doses. The TG protocol was formulated to mimic standard Intermittent Hypoxic Training (IHT) models [30], using longer hypoxic bouts to induce a steady decline in oxygen saturation, which is the classic trigger for HIF-1α adaptations [31]. In contrast, the CG protocol was specifically designed using a highly fragmented approach, in which rapid fluctuations maximize ROS-mediated signaling without excessive hypoxemic stress, in accordance with the principles of the Normobaric Hypoxia Paradox [23,24]. Finally, the hyperoxic periods were selected to accelerate hemoglobin resaturation and metabolic recovery in order to amplify the redox signaling cascade while attenuating overall cardiovascular strain during exercise [19].

At the beginning of the experiment (T0), saliva, urine, and blood samples were collected as baseline measurements. Saliva and urine samples were collected every two workouts, specifically at the end of the second workout of each week, corresponding to the first (T1), second (T2), third (T3), and final (T4) weeks. At T4, blood samples were also collected. During and after each session, cardiorespiratory parameters (HR and SpO_2_) were recorded, and the BORG scale and Lake Louise test (2018, Roach) for fatigue evaluations and Acute Mountain Sickness (AMS) were administered.

All subjects provided written informed consent (ICF) before inclusion in the study. The study was conducted in accordance with the Declaration of Helsinki and was approved by the Ethics Committee of the Provinces of Chieti and Pescara, Italy (document n. 18, 29 July 2021).

### 2.2. Subjects

A group of 12 healthy volunteers, 6 males and 6 females (29.83 ± 3.78 years old, 24–35 95% I.C.; 22.44 ± 1.80 kg/m^2^, 19–25.25 95% I.C.), was recruited and assigned without randomization to the two study groups in a balanced manner. See Table 1 for more details.

### 2.3. Inclusion Criteria

To be eligible for participation in this study, subjects needed to meet the following requirements:

- >18 years old.
- Moderate physical activity (minimum 2 times/week).
- Good exercise tolerance (assessed qualitatively via clinical interview, defined as the ability to perform without interruption-programmed physical activity).

### 2.4. Exclusion Criteria

Subjects were excluded from the study if they met any of the following criteria:

- Presence of significant chronic pathologies (e.g., cardiovascular, respiratory, or metabolic disorders).
- Abnormal routine laboratory values, including hematological alterations (e.g., anemia), metabolic profile disturbances, impaired glucose regulation or elevated inflammatory markers.
- Current use of long-term pharmacological treatments.
- Failure to meet the minimum threshold of two aerobic workouts per week.
- Professional athletes.

### 2.5. The Machine

The hypoxia–hyperoxia machine consisted of a prototype OX2 Master (Z-Health s.r.l., Noventa, Vicenza, Italy) already validated as safe for medical use. A compressor is connected to a membrane that enriches or purifies the ambient air from oxygen. The delivery of a correct FiO_2_ was confirmed by an electrode positioned on the outlet tube. The maximal airflow of the ventilator was 24 L/min in hyperoxia, 30 L/min in hypoxia and 35 L/min during normoxia. Air filters were used to improve air purity, and CO sensors were set to a limit of 10 ppm.

Subjects wore a custom, 3D-printed airtight silicon face mask covering the nose and mouth, secured by a velcro strap to ensure a tight fit. The mask featured two unidirectional valves designed to minimize dead space to only the internal mask and airway volume. This configuration prevented rebreathing by ensuring that expired air was dispersed entirely outside the circuit, thus avoiding CO_2_ retention.

**Figure 2:**
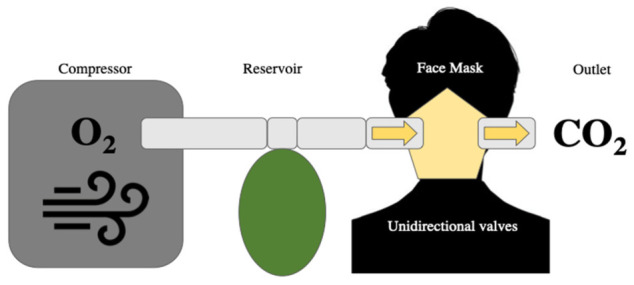
Schematic of the respiratory circuit. The system comprises a compressor delivering a constant gas mixture (hypoxic, normoxic, or hyperoxic) via a reservoir bag to a 3D-printed face mask covering nose and mouth. Unidirectional valves ensure CO_2_ is expelled directly into the environment, minimizing dead space and preventing rebreathing.

A 2 L rubber balloon served as a reservoir, as the ventilator operated in continuous mode rather than responding to a patient’s trigger. In the event of increased respiratory effort, either the compressor power or the reservoir volume would need to be adjusted to accommodate the higher minute ventilation. The machine was optimized just for low-intensity training. We opted for a smaller reservoir to maintain precise control over the FiO_2_; while a larger balloon would have delayed stabilization of the gas mixture, the 2 L volume ensured that the transition was completed within three to five breaths. Figure 2 reports the graphical details.

### 2.6. Cardiovascular and Perceived Exertion and Symptomatology Assessment

During the experiment and after each session, the lowest SpO_2_ and highest Heart Rate values were recorded via continuous pulse oximetry (Oxy 50, GIMA, Milan, Italy) once the signal remained stable for at least 10 s. Perceived Exertion was assessed using the CR-10 Borg scale. Participants received instructions before each session, and the scale was administered at the end of each session. Additionally, the severity of symptoms related to Acute Mountain Sickness (AMS) was assessed using the Lake Louise Score (Roach, 2018) [8].

### 2.7. Blood, Urine, and Saliva Sample Collection

Subjects were instructed on standardized collection protocols (e.g., no food, drink, or oral medication for 1 h before sampling).

The timing of sample collection is illustrated in Figure 1. Approximately 9 mL of venous blood was drawn from the antecubital vein with subjects in a seated or supine position. Samples for oxidative stress assessment were collected into lithium–heparin and EDTA tubes (Vacutainer, Becton Dickinson, Franklin Lakes, NJ, USA). Plasma was separated by centrifugation (5702R, Eppendorf, Hamburg, Germany) at 3500× *g* for 10 min at 4 °C. Aliquots of plasma and erythrocytes were then stored at −80 °C until analysis. To avoid degradation, samples were thawed only once and analyzed within one month of collection.

Urine was collected via voluntary voiding into sterile containers and stored in aliquots at −20 °C until assayed. Approximately 1 mL of saliva was collected using Salivette devices (Sarstedt, Nümbrecht, Germany). Salivettes were centrifuged at 1500× *g* for 20 min at 4 °C; the retrieved saliva was then aliquoted and stored at −80 °C until analysis, with only a single freeze–thaw cycle permitted.

A complete blood count was performed to ensure all participants were within healthy clinical ranges; however, only the parameters relevant to the study objectives are reported here. Venous blood samples were collected for standardized clinical hematological and biochemical analyses. The following parameters were determined using an automated analyzer (cobas^®^, Basel, Switzerland), according to standard laboratory methods: reticulocytes (Ret), absolute reticulocyte count (Af), immature reticulocytes fraction (Im), glucose (Glu), triglycerides (Tri), high-density lipoprotein (HDL), total cholesterol (Tc), low-density lipoprotein (LDL), apolipoprotein A1/B ratio (Apo A1/B), estimated glomerular filtration rate (eGFR), and Erythrocyte Sedimentation Rate (ESR).

### 2.8. Oxidative Stress and Antioxidant Levels

The Reactive Oxygen Species (ROS) production rate was quantified by Electron Paramagnetic Resonance (EPR) spectroscopy. An X-band EPR spectrometer (9.3 GHz) (E-Scan, Bruker Co., Billerica, MA, USA) was used to detect the ROS production rate in saliva samples at 37 °C using a Temperature Controller unit (Noxigen Science Transfer & Diagnostics GmbH, Elzach, Germany). The spin probe CMH (1-hydroxy-3-methoxy-carbonyl-2,2,5,5-tetramethylpyrrolidine) was utilized, while the stable radical CP (3-carboxy-2,2,5,5-tetramethyl-1-pyrrolidinyloxy) served as an external reference to convert EPR signals into absolute quantitative values (µmol/min) [32].

Total Antioxidant Capacity (TAC) was measured in saliva using a commercial kit (Item No. 709001, Cayman Chemical, Ann Arbor, MI, USA) according to established protocols [21]. Briefly, 10 µL of saliva was added in duplicate to 10 μL of metmyoglobin and 150 μL of the chromogen solution. Reactions were initiated by adding 40 μL of H_2_O_2_, incubated at room temperature for 3 min, and read at 750 nm.

Lipid peroxidation was assessed in urine by measuring 8-isoprostane concentration (8-iso-PGF2α; Item No. 516360, Cayman Chemical, Ann Arbor, MI, USA) via a competitive immunoassay. Concentrations were determined using a standard curve, with samples and standards read at 412 nm, as previously described [33].

### 2.9. Inflammatory Status

Interleukins IL-6 and IL-10 levels were determined in saliva using ELISA kits (IL-6: No. 501030, Cayman Chemical, Ann Arbor, MI, USA; IL-10: Cat. No. EH0173, Fine Test, Wuhan, China) following the manufacturers’ instructions and previously described methods [34].

### 2.10. Renal Function Assessment

Urinary creatinine, neopterin, and uric acid concentrations were measured via High-Performance Liquid Chromatography (HPLC) as described by Vernerová [35], using a Varian instrument (pump 240, autosampler ProStar 410, Palo Alto, CA, USA). Detection was performed with a UV-VIS detector (Shimadzu SPD 10-AV, Kyoto, Japan, λ = 240 nm for creatinine and uric acid; JASCO FP-1520, Tokyo, Japan, λex = 355 nm and at λem = 450 nm) for neopterin. Following centrifugation at 13,000 rpm for 5 min at 4 °C, analytic separations were performed at 50 °C on a 5 µm Discovery C18 analytical column (250 × 4.6 mm I.D., Supelco, Sigma-Aldrich, St. Louis, MO, USA) at a flow rate of 0.9 mL/min. Calibration curves were linear over the range of 0.125–1 µmol/L for neopterin, 0.625–20 mmol//L for uric acid and 1.25–10 mmol/L for creatinine. The inter-assay and intra-assay coefficients of variation were <5%.

### 2.11. Statistical Analysis

Data were analyzed using Linear Mixed Models (LMMs), which are appropriate for longitudinal repeated measures designs and small sample sizes. In the models, the participant ID was included as a random factor with a random intercept to account for individual baseline differences. The fixed-effects structure included the condition (TG vs. CG) as a categorical factor, the number of sessions as a continuous covariate, and their interaction (condition × training), allowing for the assessment of both group differences and differential trajectories over time. A variance components covariance structure was specified, assuming the independence of the random effects across subjects and accounting for within-subject correlation through the inclusion of a random intercept.

Although biological adaptations to training are inherently non-linear, modeling training exposure as a continuous covariate was intentionally adopted to preserve degrees of freedom and maintain statistical power given the limited sample size and number of repeated measurements. This approach is commonly recommended when the number of time points is small and the primary aim is to estimate the overall trajectory of change rather than to model complex non-linear patterns [36,37]. More complex non-linear or distribution-specific models would require larger sample sizes to ensure stable parameter estimation and to reduce the risk of overfitting. For previous reasons, the β coefficients derived from the models should be interpreted as estimates of the average linear trend across the intervention period, rather than precise representations of the underlying biological kinetics.

Model selection was guided by residual diagnostics rather than by the raw distribution of the variables. Specifically, residual assumptions were evaluated through the visual inspection of Q–Q plots, histograms of residuals, and residuals versus fitted values plots; this approach was preferred over formal normality tests (Shapiro–Wilk), as such tests are known to be unreliable in small samples, either lacking power or being overly sensitive to minor deviations, thus potentially leading to misleading conclusions or unnecessary data transformations [38].

Statistical significance was set at *p* < 0.05. All statistical analyses were performed using Jamovi software (2.3.28), and figures were set through GraphPad Prism (11.0.0).

## 3. Results

### 3.1. Safety and Adherence

Participants demonstrated 100% adherence to the training protocol, and no severe adverse events requiring medical intervention or dropout were recorded. Safety outcomes, specifically Acute Mountain Sickness (AMS), were monitored quantitatively using the Lake Louise Score (LLS). During the first and second workouts, two cases of moderate AMS (LLS = 5) were observed, distributed equally between groups (*n* = 1 in TG; *n* = 1 in CG). By the third workout, the clinical trajectories changed; the CG participant’s symptoms decreased to mild AMS, whereas the TG participant maintained a moderate AMS score (LLS = 5). Subsequently, these symptoms fully resolved in both groups as acute acclimatization occurred, with the sole exception of the sixth session, where moderate AMS (LLS = 5) transiently reappeared in the same TG subject before disappearing completely. The overall symptoms reported during training were mild and resolved within hours post-exposure. In particular, the occurrence of headache was recorded in 25% (*n* = 3) of participants during the first session, 33.3% (*n* = 4) during the second session, 16.6% (*n* = 2) during the third session and 8.3% (*n* = 1) during the sixth session. By group, these headache incidences were as follows: TG (*n* = 2 in session 1; *n* = 2 in session 2; *n* = 1 in session 3; and *n* = 1 in session 6) and CG (*n* = 1 in session 1; *n* = 2 in session 2; *n* = 1 in session 3; and *n* = 0 in session 6).

### 3.2. Cardiovascular Adaptations

Regarding SpO_2_ (R_2_m = 0.050), no significant main effect of training was observed (F(1, 77.9) = 0.02, *p* = 0.877, β = 0.03), indicating no overall alteration over time, and no significant main effect was found for conditioning (F(1, 10.0) = 0.62, *p* = 0.450, β = 1.62). However, a significant conditioning*training (C × T) interaction was found (F(1, 77.9) = 4.05, *p* = 0.048, β = 0.69). This interaction indicates that hyperoxic conditioning significantly attenuated the decline in SpO_2_ observed during the TG compared to the CG.

In contrast, maximal Heart Rate (HR) did not show a significant main effect for training (F(1, 76.7) = 0.24, *p* = 0.624, β = −0.18), suggesting that the protocol did not systematically alter HR across sessions. However, while the model did not achieve statistical significance for conditioning (F(1, 10.1) = 3.25, *p* = 0.101, β = 11.99) or the C × T training interaction (F(1, 76.7) = 3.09, *p* = 0.080, β = −1.38), the β estimates suggest a potential distinct physiological trajectory. In fact, the negative interaction coefficient (β = −1.38) indicates that the hyperoxic group (CG) exhibited a more stable, or slightly lower, Heart Rate progression over time.

**Figure 3:**
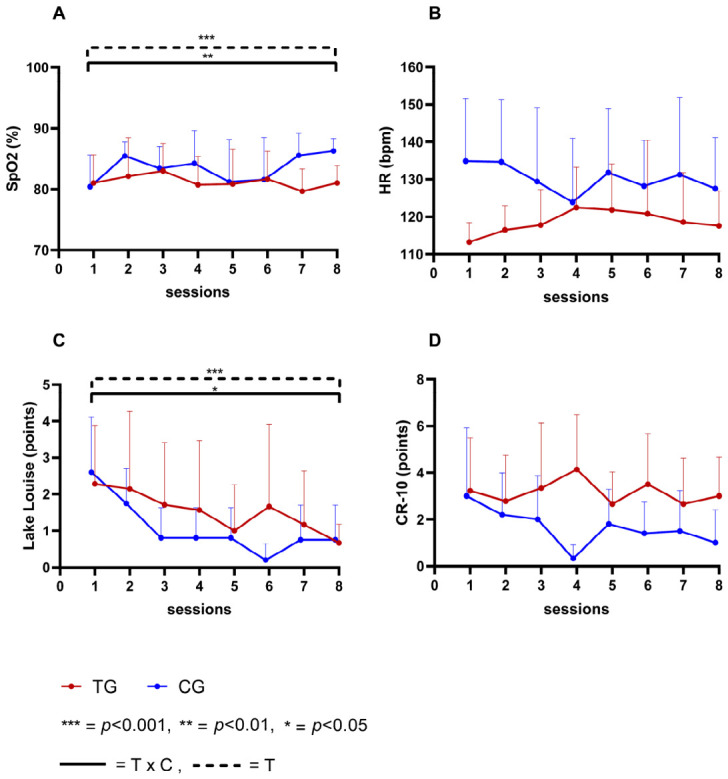
Physiological and perceptual responses across the 4-week IHHT intervention. Data are shown for the Training Group (TG) and Conditioning Group (CG): (**A**) Peripheral oxygen saturation (SpO_2_), (**B**) Heart Rate (HR), (**C**) Perceived Exertion (CR-10), and (**D**) Lake Louise Score ([8]) across sessions. Abbreviations: T: main effect of time (sessions); C × T: interaction effect between time and condition. Data are expressed as mean ± SD.

Figure 3 shows the mean ± SD values of peripheral oxygen saturation (SpO_2_) and Heart Rate (HR) across the training sessions for both groups, while the detailed results of the mixed-effects models are presented in Table 1.

### 3.3. Perceived Exertion and Symptomatology

Regarding subjective assessments, the Lake Louise Score used to evaluate Acute Mountain Sickness symptoms (R_2_m = 0.149) showed no significant differences related to conditioning (F(1, 10.1) = 0.59, *p* = 0.45, β = −0.50). In contrast, statistically significant differences emerged regarding training (F(1, 77.5) = 29.16, *p* < 0.001, β = −0.23), but no regarding C × T interaction (F(1, 77.5) = 0.23, *p* = 0.626, β = 0.04). A different pattern emerged for the Borg scale (R_2_m = 0.143), which no difference emerged between the two conditions (F(1, 9.8) = 2.75, *p* = 0.129, β = −1.59) or over time (F(1, 74.5) = 2.89, *p* = 0.093, β = −0.10), but stastically differences was found in the C × T interaction ((F(1, 74.5) = 4.91, *p* = 0.03, β = −0.27). Figure 3 shows the mean ± SD values of Borg Perceived Exertion (CR-10) and Lake Louise Score across the training sessions for both groups, while the detailed results of the mixed-effects models are presented in Table 2.

### 3.4. Biochemical Parameters

**Figure 4:**
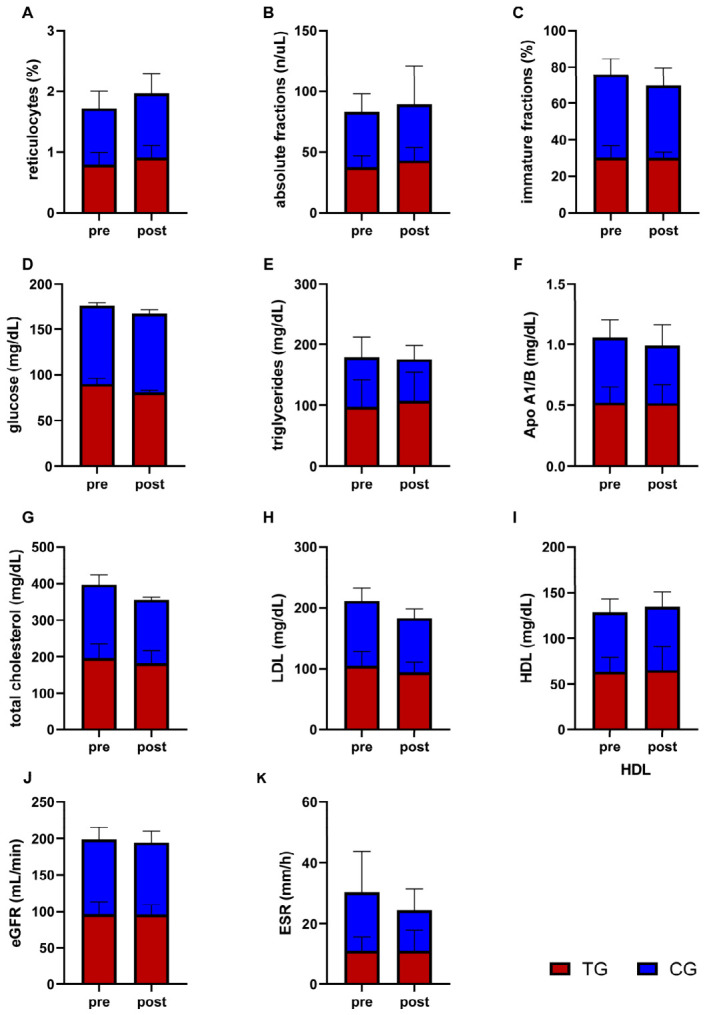
Biochemical and hematological parameters across the 4-week IHHT intervention. Data are presented for the Training Group (TG) and Conditioning Group (CG). Abbreviations: T: main effect of time (sessions); T × C: interaction effect between time and condition. Panels: (**A**) reticulocytes percentage, (**B**) absolute fractions, (**C**) immature reticulocyte fraction, (**D**) glucose, (**E**) triglycerides, (**F**) Apo A1/B ratio (apolipoprotein A1/apolipoprotein B), (**G**) total cholesterol, (**H**) LDL (low-density lipoprotein cholesterol), (**I**) HDL (high-density lipoprotein cholesterol), (**J**) eGFR (estimated glomerular filtration rate), and (**K**) ESR (Erythrocyte Sedimentation Rate).

No significant effects were found regarding reticulocytes (Ret) R_2_m = 0.0955), absolute fraction (Af) (R_2_m = 0.0974), or immature reticulocytes fraction (Im) (R_2_m = 0.475). Specifically, no differences were observed based on conditioning (Ret: F(1, 8.2) = 0.5, *p* = 0.867, β = 0.0969; Af: F(1, 9.4) = 0.6, *p* = 0.867, β = 5.779; Im: F(1, 6.4) = 16.6, *p* = 0.066, β = 12.61), training (Ret: F(1, 7.1) = 1.9, *p* = 0.374, β = 0.015; Af: F(1, 7.6) = 2.5, *p* = 0.334, β = 0.841; Im: F(1, 6.3) = 1.0, *p* = 0.445, β = −0.381), or the C × T interaction (Ret: F(1, 7.1) = 0.007, *p* = 0.933, β = 0.001; Af: F(1, 7.6) = 0.1, *p* = 0.886, β = 0.357; Im: F(1, 6.3) = 0.9, *p* = 0.866, β = −0.729). Glucose (R_2_m = 0.410) showed no differences between the two conditions (F(1, 18) = 0.004, *p* = 0.999, β = 0.127), training (F(1, 18) = 5.6, *p* = 0.267, β = −0.556) and the T × C interaction (F(1, 18) = 6.2, *p* = 0.242, β = 1.174). Triglycerides were not affected (R_2_m = 0.0575; conditioning F(1, 9.8) = 0.7, *p* = 0.867, β = −19.442; training F(1, 8.07) = 0.06, *p* = 0.800, β = 0.224; T × C F(1, 8.07) = 0.25, *p* = 0.886, β = −0.860), and neither was HDL (R_2_m = 0.008; conditioning F(1, 10.2) = 0.05, *p* = 0.999, β = 2.339; training F(1, 8.4) = 0.5, *p* = 0.485, β = 0.297; T × C F(1, 8.4) = 0.06, *p* = 0.886, β = 0.211). Total cholesterol (TC) and LDL did not achieve statistical significance during training, although the negative β estimates indicate a quantitative reduction in these parameters (TC: F(1, 8.9) = 3.4, *p* = 0.267, β = −2.372; LDL: F(1, 8.7) = 3.8, *p* = 0.267, β = −1.565). No differences for these parameters were observed across conditioning (TC: F(1, 10.0) = 9.37 × 10^−4^, *p* = 0.999, β = 0.501; LDL: F(1, 9.8) = 2.13 × 10^−6^, *p* = 0.999, β = −0.001) or the T × C interaction (TC: F(1, 8.9) = 0.1, *p* = 0.886, β = −0.970; LDL: F(1, 8.7) = 0.06, *p* = 0.886, β = −0.404). Similarly, the Apo A1/Apo B ratio (R_2_m = 0.028) showed no differences regarding conditioning (F(1, 10.1) = 0.01, *p* = 0.999, β = 0.010), and while statistical significance was not reached across training (F(1, 7.4) = 3.5, *p* = 0.267, β = −0.004) or the C × T interaction (F(1, 7.4) = 3.3, *p* = 0.611, β = −0.009) the β values reflect a slight negative shift. Finally, no significant differences emerged regarding eGFR (R2m = 0.060; conditioning F(1, 9.9) = 0.6, *p* = 0.867, β = 7.699; training F(1, 8.0) = 1.3, *p* = 0.445, β = −0.211; T × C F(1, 8.0) = 1.0, *p* = 0.886, β = −0.381) and ESR (R_2_m = 0.155; conditioning F(1, 7.1) = 2.4, *p* = 0.867, β = 7.230; training F(1, 6.0) = 0.3, *p* = 0.641, β = −0.185; T × C F(1, 6.0) = 0.2, *p* = 0.886, β = −0.322). Figure 4 shows the mean ± SD values of all biochemical parameters across the training sessions for both groups, while the detailed results of the mixed-effects models are presented in Table 3. Table A1 in Appendix A presents the mean ± SD (standard deviation) of biochemical panel parameters measured before (Pre) and after (Post) the IHHT protocol.

### 3.5. Oxidative Stress and Inflammation

Reactive Oxygen Species (ROS) production significantly increased during the training protocol (R_2_m = 0.528, F(1, 45.4) = 62.5, *p* < 0.001, β = 0.122). No significant effects were observed for conditioning (F(1, 10.2) = 1.1, *p* = 0.517) or the C × T interaction (F(1, 45.4) = 0.09, *p* = 0.756, β= 0.009), indicating that the increase in ROS was independent of the oxygen concentration used. Conversely, the Total Antioxidant Capacity (TAC) exhibited a significant progressive reduction through the training (R_2_m = 0.245, F(1, 44.3) = 26.2, *p* < 0.001, β = −0.099). Similarly, 8-isoprostane (8-ISO) levels significantly rose over the training sessions (R_2_m = 0.473, F(1, 44.4) = 50.3, *p* < 0.001, β = 68.7). Notably, the C × T interaction did not reach statistical significance for either TAC (F(1, 44.3) = 1.5, *p* = 0.376) or 8-ISO (F(1, 44.4) = 2.18, p = 0.376).

**Figure 5:**
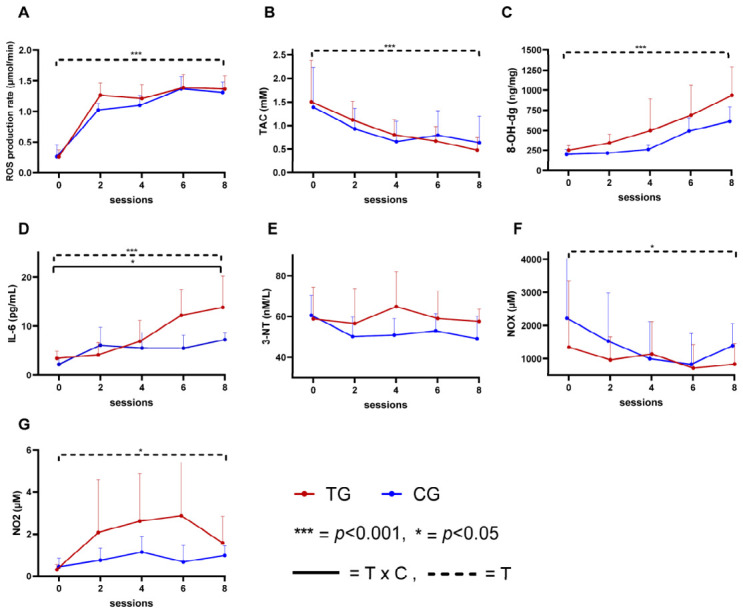
Oxidative stress and inflammatory salivary markers across the 4-week IHHT intervention. Data are presented for the Training Group (TG) and Conditioning Group (CG). Abbreviations: T: main effect of time (sessions); C × T: interaction effect between time and condition. Panels: (**A**) ROS (Reactive Oxygen Species), (**B**) TAC (Total Antioxidant Capacity), (**C**) 8-ISO (8-isoprostane), (**D**) IL-6 (Interleukin-6), (**E**) 3-NT (3-Nitrotyrosine), (**F**) NOx (total Nitric Oxide metabolites), and (**G**) NO_2_ (Nitrite). Data are expressed as mean ± SD.

Regarding nitrosative stress, 3-Nitrotyrosine (3-NT) showed no significant changes related to training (F(1, 44.0) = 1.5, *p* = 0.220), conditioning (F(1, 9.7) = 0.8, *p* = 0.517), or their interaction (F(1, 44.0) = 0.5, *p* = 0.544, R_2_m = 0.057). Interleukin-6 (IL-6) demonstrated a robust response to the protocol (R_2_m = 0.473). A significant main effect of training indicated a general increase in inflammatory status across protocol (F(1, 44.7) = 41.1, *p* < 0.001, β = 0.978). Interestingly, a highly significant C × T interaction was observed (F(1, 44.7) = 9.9, *p* = 0.021, β = −0.961). Nitric Oxide total metabolite (NOx: NO_2_ + NO_3_) levels significantly decreased across the training protocol (R_2_m = 0.075, F(1, 44.3) = 7.0, *p* = 0.015, β = −99.2), but no effect emerged regarding conditioning (F(1, 10.1) = 0.4, *p* = 0.595), and interaction (F(1, 44.3) = 0.7, *p* = 0.544). Finally, Nitrite (NO_2_) levels showed a significant increase during training (R_2_m = 0.163, F(1, 44.0) = 5.1, *p* = 0.034, β = 0.137), with no significant differences found for conditioning (F(1, 9.6) = 2.3, *p* = 0.359) or interaction (F(1, 44.0) = 1.7, *p* = 0.376). Figure 5 shows the mean ± SD values of oxidative stress and inflammation parameters across the training sessions for both groups, while the detailed results of the mixed-effects models are presented in Table 4.

### 3.6. Renal Status

Uric acid (AU) levels remained stable throughout the study protocol (R_2_m = 0.079), with no significant effects observed during training protocol (F(1, 44.4) = 0.004, *p* = 0.949) or conditioning (F(1, 9.9) = 2.1, *p* = 0.513). Similarly, the C × T interaction was not significant (F(1, 44.4) = 0.2, *p* = 0.627), indicating that the uric acid was unaffected by the type of oxygenation protocol used.

**Figure 6:**
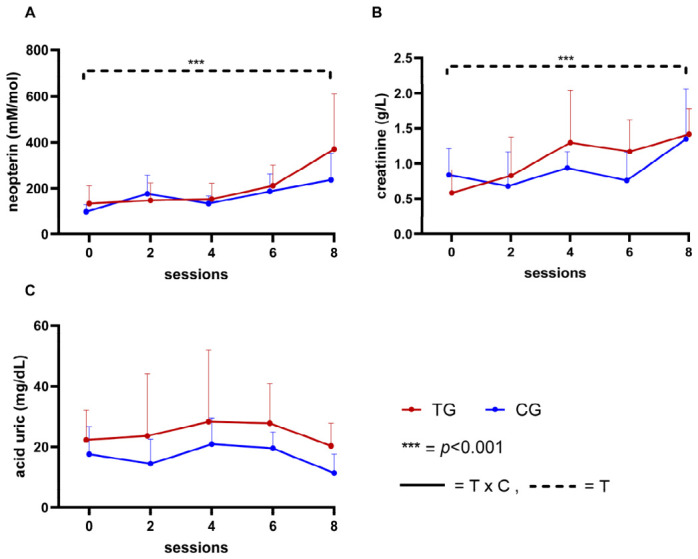
Renal function and immune response markers across the 4-week IHHT intervention. Data are presented for the Training Group (TG) and Conditioning Group (CG). Abbreviations: T: main effect of time (sessions); C × T: interaction effect between time and condition. Panels: (**A**) neopterin, (**B**) creatinine, (**C**) uric acid. Data are expressed as mean ± SD.

In contrast, creatinine (Crea) showed a different pattern (R_2_m = 0.193). While a highly significant effect was observed during training sessions (F(1, 44.6) = 14.2, *p* < 0.001, β = 0.07), the C × T interaction did not reach significance (F(1, 44.6) = 1.8, *p* = 0.309). Similarly, neopterin (Neopt) levels increased sharply in response to training (R_2_m = 0.244, F(1, 44.3) = 27.5, *p* < 0.001, β = 19.2); however, no significant differences were found between conditions (F(1, 10.1) = 0.4, *p* = 0.513), or for the interaction (F(1, 44.3) = 2.7, *p* = 0.309). Figure 6 shows the mean ± SD values of renal function and immune response parameters across the training sessions for both groups, while the detailed results of the mixed-effects models are presented in Table 5.

## 4. Discussion

This Exploratory Pilot Study aimed to explore the safety and feasibility of two distinct Intermittent Hypoxic–Hyperoxic Training (IHHT) protocols through preliminary physiological responses. To the best of our knowledge, this work is the first to evaluate physiological, metabolic, and pro-inflammatory responses when IHHT is applied concurrently with physical exercise. The primary finding of this work is that both protocols resulted in feasible and safe outcomes, with the more balanced hyperoxic–hypoxic exposure (CG) demonstrating superior acute physiological tolerability compared to the unbalanced exposure (TG). As secondary exploratory findings, we observed that the CG protocol seemed to attenuate cardiovascular load and systemic inflammation, and we noted preliminary trends in lipid parameters, highlighting distinct physiological adaptations that warrant further investigation. Currently, the effects of IHHE on total cholesterol, high- and low-density lipoprotein cholesterol levels, as well as erythropoiesis and hemoglobin mass are still inconclusive [7]. The existing literature predominantly highlights the benefits of IHHT, such as improved cardiorespiratory fitness, cognitive, and functional capacity, almost exclusively in clinical populations [16,17,18,19]. Furthermore, metabolic improvements observed in IHHT have been primarily conducted in the presence of dysmetabolic patients [39]; in these cohorts, when compared with control groups performing exercise alone, IHHT often fails to demonstrate superior benefits, suggesting that the metabolic stress of exercise might be the primary driver of adaptation. While hematological adaptations were not observed in our study, our data showed a consistent negative β for TC, LDL, and the Apo A1/Apo B ratio in the CG. Although these parameters did not reach statistical significance after the conservative Benjamini–Hochberg correction for multiple comparisons and probably due to the reduced sample size typical of a pilot study, the direction of the effect β suggests a potential modulation of the lipid profile. Rather than drawing definitive conclusions, this observed trend highlights a potential area for future research, especially in healthy subjects, where optimizing exercise for primary prevention may represent a novel strategy. It remains to be investigated whether the specific hypoxic–hyperoxic ‘dose’ and intensity applied in our protocol might interact with lipid signaling pathways differently than exercise alone or IHH interventions [28]. While our non-significant data preclude definitive conclusions, the existing literature provides a theoretical rationale for these trends, suggesting that hypoxic–hyperoxic exposure may suppress lipogenic regulators (e.g., SREBP-1c) [40] and stimulate HIF-driven lipid clearance and oxidation [41,42,43,44].

An interesting result is the protective physiological effect shown in the Conditioning Group (CG). While a reduction in SpO_2_ is expected under hypoxic exposure, participants affected by an optimal hypoxic–hyperoxic ratio demonstrated a significantly attenuated decline in peripheral oxygen saturation (SpO_2_) across sessions (*p* = 0.048), demonstrating either adaptation or minor stress. These results are of significant clinical interest for evaluating the dose–response relationship, particularly because the existing literature identifies decreased SpO_2_ during exercise as a critical risk factor for the onset of Acute Mountain Sickness (AMS) [45,46]. Similarly, the negative β observed in the HR of CG compared to TG could indicate a reduced myocardial oxygen demand and consequently a lower myocardial workload and cardiovascular stress in this exploratory setting [47,48]. Supporting what we previously discussed, the perception of effort also shows a significantly different response pattern. While overall CR-10 scores did not change over the sessions across both groups (*p* = 0.093), the kinetic response was different. In fact, CG showed a significant reduction in perceived exertion (*p* = 0.03). Except for two cases of Acute Mountain Sickness and some reports of headache and fatigue during, no further important adverse events were observed, resulting in a safe, well-tolerated, and effective program for all participants.

Our observations suggest that a 4-week IHHT regimen induces a pronounced pro-oxidant state, characterized by a cumulative increase in ROS production rate (*p* < 0.001), lipids peroxidation (8-iso-PGF2a; *p* < 0.001) and a concomitant reduction in Total Antioxidant Capacity (*p* < 0.001). This redox shift functions as a primary trigger for redox-sensitive signaling pathways. Specifically, the existing literature demonstrates that the transient rise in ROS [49] induces the phosphorylation of p38 MAPK and the nuclear translocation of NF-kB [50]. Although these specific intracellular pathways were not directly measured in our study, we hypothesize that this molecular cascade likely acts as a double-edged sword. Crucially, this signaling works coordinately with the Nrf2 pathway; as a result, those transcription factors can organize a hormetic program that upregulates key endogenous antioxidant enzymes [51]. This proposed mechanism is consistent with the Normobaric Oxygen Paradox, whereby O_2_-dependent oscillations in ROS contribute to the activation of PGC-1α and mitochondrial biogenesis [52]. In addition, they have been shown by previous studies to be involved in the release of pro-inflammatory mediators such as IL-6 and neopterin, providing a mechanistic rationale for the changes in these specific mediators observed in our cohort [53].

Salivary Interleukin-6 levels increased in all subjects following the initial sessions (*p* < 0.001), reflecting the acute systemic stress and the activation of the endocrine-immune pathways induced by exercise. However, the CG exhibited a significantly attenuated response across protocol training (*p* = 0.021). IL-6 is a pleiotropic cytokine involved in immunity, tissue regeneration and various pathologies [54]. During exercise, it acts as a marker of the systemic response and a mediator of hormetic responses [55]. In this context, the observed reduction in IL-6 over time suggests protective hormetic adaptation, enhances the organism’s resilience to exercise stress, and modulates the pro-inflammatory signaling cascade. Although hypoxia [33] and hyperoxia have been shown to increase IL-6 [19,21,25,33], when associated with exercise, hypoxia and hyperoxia elicit different responses. Hypoxic exercise does not increase IL-6 more compared to exercise alone [33]; on the other hand, hyperoxic chronic exercise increases IL-6 production [23] while pre-exposure to hyperoxic air before exercise does not influence IL-6 after 17 sessions [56]. Importantly, the degree of elevation of IL-6 after hyperoxic exercise is dependent on multiple factors, such as exercise intensity, the duration of exercise, and an individual’s exercise capacity [57]. Although the increase in IL-6 after 5 weeks of hyperoxic training was attributed to oxygen’s role in increasing metabolic load and, consequently, the hormetic response that enhances exercise benefits [23], it is well established that IL-6 transcription is primarily regulated by the activation of NF-κB [58]. In this regard, we propose that the CG protocol shifts the IL-6 response toward an adaptive, ‘myokine-like’ profile by mitigating the excessive oxidative stress observed in the TG. Because IL-6 acts as a myokine under mechanical load but as an inflammatory cytokine under systemic stress, this modulation likely reflects a more favorable redox signaling environment. Our results suggest that a balanced ratio of hyperoxia, normoxia, and hypoxia promotes the overexpression of antioxidant enzymes. This, in turn, reduces the systemic inflammatory response, consistent with the findings reported by Bosco et al. [20] in the absence of exercise.

Finally, creatinine levels increased following repeated exercise training (*p* < 0.001). In fact, exercise typically increases creatinine levels in proportion to intensity [59] and induces reductions in renal blood flow and eGFR, representing acute renal stress [40]. Hypoxia may exacerbate this pattern [21,60], potentially leading to renal dysfunction. At the same time, hyperoxia has also been shown to increase these markers [23]. Therefore, the observed elevations in creatinine and neopterin should be interpreted as a composite outcome of exercise-induced metabolic and mechanical stress rather than direct renal or immune pathology, with all values remaining within clinically healthy ranges throughout the study.

This supports the hypothesis that hyperoxia might not blunt the fundamental exercise-induced adaptations but rather refines the hormetic response, thereby triggering a multi-organ protective mechanism [28].

### Limitations

This study presents several limitations. First, while the small sample size is appropriate for an early-stage feasibility and safety study, the statistical power is insufficient to assess rigorously the comprehensive panel of highly fluctuating oxidative stress and biochemical markers. Although we applied False Discovery Rate (FDR) corrections to mitigate the risk of Type I errors from multiple comparisons, the small sample size (*N* = 12) inherently increases the risk of Type II errors and remains insufficient to fully account for high inter-individual variability. Moreover, the absence of a normoxic control group limits the interpretation of any efficacy-related outcomes. Without this baseline comparison, it is impossible to draw definitive causal inferences regarding the specific physiological efficacy of the hypoxic stimulus independent of the exercise intervention itself. However, it is important to note that since both the Training Group (TG) and the Conditioning Group (CG) performed an identical exercise training, the observed between-group differences in the metabolic and inflammatory trajectories are attributable to the IHHT protocols rather than the exercise itself. This comparative approach effectively isolates the specific impact of hypoxic–hyperoxic modulation, partially mitigating the limitations inherent in non-controlled exercise settings. For these reasons, the findings reported should be considered strictly preliminary and confirmed by future controlled studies.

Secondly, the standardization of the exercise load could have been better tailored using metabolic-based methods or a prior stress test. Individualizing the training intensity and the hypoxic dose based on each participant’s basal physiological responses would help reduce potential bias and allow for subjects to be compared in a more rigorous setting. This aspect will certainly be addressed in future applications of these protocols.

Moreover, although low exercise intensity was associated with increased Hb [24], using mild hyperoxia (30% O_2_) instead of 100% oxygen allows us to reduce ROS production but can affect the hematological responses observed by Balestra et al. [23].

Another limitation concerns the assessment of arterial oxygen saturation (SpO_2_). Although a stabilization period of at least 10 s was strictly respected and the Tannheimer et al. recommendations were observed [61], the analysis used the lowest stable recorded SpO_2_ rather than the mean SpO_2_ for the entire exercise session. This approach was chosen to capture the peak acute physiological challenge. As noted by Dünnwald et al. [62], the reliability of these devices can be compromised at lower saturation levels, especially under hypoxic conditions. Consequently, while the lower value reflects the most pronounced response, it may also involve a higher degree of inherent variability compared to a session-wide average.

Participants’ dietary habits were not standardized or recorded during the intervention period. Given that nutritional intake can significantly modulate lipid and metabolic profiles, its unmeasured influence remains a limitation of this study, underlining the need to incorporate dietary monitoring in subsequent investigations.

## 5. Conclusions

In conclusion, this Exploratory Pilot Study demonstrates that a 4-week IHHT program is a feasible and well-tolerated approach in a healthy cohort. The simultaneous combination of IHH and exercise suggests that a balanced hyperoxic-to-hypoxic ratio appears to modulate the training stimulus by attenuating cardiovascular strain and mitigating systemic inflammation, specifically reducing IL-6. Moreover, the absence of significance in other cytokines and the preliminary variations noted in lipid profiles highlight an interesting trend for future research. From these preliminary effect sizes, future studies are required to isolate the effects of the IHHT from the normal physiological responses induced by the exercise to confirm these preliminary findings.

## Figures and Tables

**Table 1:** Participant characteristics. TG = Training Group; CG = Control Group; and BMI = Body Mass Index. F/W = frequency of weekly training sessions (number of training sessions performed per week).

Subjects Group	Years Old	Weight (kg)	High (cm)	BMI (m^2^/kg)	F/W	Typologies
TG 1	28	67	170	23.18	≥3 ≤ 5	Running
TG 2	25	53	166	19	≥3 ≤ 5	Athletics
TG 3	35	72	178	22.7	≥3 ≤ 5	Running
TG 4	35	82	190	22.48	≥3 ≤ 5	Triathlon
TG 5	32	78	176	24.9	≥3 ≤ 5	Running
TG 6	29	80	177	25.25	≥3 ≤ 5	Crossfit
TC 1	31	56	164	20.82	≥3 ≤ 5	Running
TC 2	24	70	172	23.39	≥3 ≤ 5	Resistance training
TC 3	34	57	160	22.27	≥3 ≤ 5	Running/Pilates
TC 4	26	75	180	23.14	≥3 ≤ 5	Resistance training
TC 5	28	68	175	21.95	≥3 ≤ 5	Crossfit
TC 6	31	64	176	20.2	≥3 ≤ 5	Running

**Table 2:** Linear Mixed-Effects Model analysis evaluating the effects of the IHHT protocol, training sessions, and their interaction on physiological and perceptual parameters. Data are presented as β estimates with 95% Confidence Intervals [Lower; Upper]. Intercept: Baseline reference value for the Training Group (TG). Conditioning: Main effect of the assigned IHHT protocol. The β estimate reflects the mean difference between the Conditioning Group (CG) and the TG. Training: Main effect of time (number of sessions). The β estimate represents the average change per training session. C × T (Interaction): Time by conditioning interaction. The β estimate indicates how the change over time in the CG differs from the change observed in the TG. Abbreviations: SpO_2_ (oxygen saturation, [%]); HR (Heart Rate, [bpm]); LL (Lake Louise Score [points]; [8]); and Borg Scale (CR-10, [points]).

Parameter	R-Squared-Marginal	Intercept	Conditioning β (Estimate) *p*-Values 95% CI [L, U]	Training β (Estimate) *p*-Values 95% CI [L, U]	T × C β (Estimate) *p*-Values 95% CI [L, U]
SpO_2_	0.050	82.15 [80.13, 84.16]	β = 1.61 *p* = 0.45 [−2.4, 5.6]	β = 0.02 *p* = 0.87 [−0.3, 0.3]	β = 0.69 *p* = 0.048 [0.01, 1.3]
HR	0.171	124.105 [117.58, 130.62]	β = 11.99 *p* = 0.101 [−1.04, 25.04]	β = −0.18 *p* = 0.624 [−0.93, 0.56]	β = −1.34 *p* = 0.080 [−2.28, 0.153]
LL	0.149	1.26 [0.61, 1.9]	β = −0.50 *p* = 0.45 [−1.7, 0.7]	β = −0.23 *p*-adj <0.001 [−0.3, −0.15]	β = −0.04 *p* = 0.62 [0.21, 0.13]
Borg Scale (CR-10)	0.155	2.55 [1.6, 3.5]	β = −1.58 *p* = 0.129 [−3.46, 0.28]	β = −0.105 *p* = 0.09 [−0.22, 0.01]	β = −0.27 *p* = 0.03 [−0.5, −0.03]

**Table 3:** Linear Mixed-Effects Model analysis evaluating the effects of the IHHT protocol, training sessions, and their interaction on biochemical and hematological parameters. Data are presented as β estimates with 95% Confidence Intervals [Lower; Upper] and FDR-adjusted *p*-values (*p*-adj). Intercept: Baseline reference value for the TG. Conditioning: Main effect of the assigned IHHT protocol. The β estimate reflects the mean difference in the CG compared to the TG. Training: Main effect of time (number of sessions). The β estimate represents the average change in the biomarker per training session. T × C: Time by conditioning interaction. The β estimate indicates how the change over time in the CG differs from the change observed in the TG. Abbreviations: Ret (reticulocytes, [%]); Af (absolute fractions, [n/uL]); Im (immature reticulocytes fractions, [%]); Glu (glucose, [mg/dL]); Tri (triglycerides, [mg/dL]); HDL (high-density lipoprotein, [mg/dL]); TC (total cholesterol, [mg/dL]); LDL (low-density lipoprotein, [mg/dL]); Apo A1/B (apolipoprotein A1/apolipoprotein B ratio, [mg/dL]); eGFR (estimated glomerular filtration rate, [mL/min]); ESR (Erythrocyte Sedimentation Rate, [mM/h]). *p*-adj: indicates the adjusted *p*-values calculated using the Benjamini–Hochberg False Discovery Rate method to correct for multiple comparisons.

Parameter	R-Squared-Marginal	Intercept	Conditioning β (Estimate) *p*-Values 95% CI [L, U]	Training β (Estimate) *p*-Values 95% CI [L, U]	T × C β (Estimate) *p*-Values 95% CI [L, U]
Ret	0.095	β = 0.894 [0.768; 1.021]	β = 0.096 [−0.155; 0.349] *p*-adj = 0.867	β = 0.015 [−0.006; 0.037] *p*-adj = 0.374	β = 0.0226 [−0.042; 0.046] *p*-adj = 0.933
Af	0.097	β = 42.9999 [35.777; 50.22]	β = 5.779 [−8.664; 20.22] *p*-adj = 0.867	β = 0.841 [−0.196; 1.88] *p*-adj = 0.334	β = 0.357 [−1.716; 2.43] *p*-adj = 0.886
Im	0.475	36.533 [33.50; 39]	β = 12.618 [6.56; 18.674] *p*-adj = 0.066	β = −0.381 [−1.13; 0.364] *p*-adj = 0.445	β = −0.729 [−2.22; 0.761] *p*-adj = 0.886
Glu	0.410	86.05 [84.221; 87.880]	β = 0.127 [−3.53; 3.786] *p*-adj = 0.999	β = −0.556 [−1.015; 0.096] *p*-adj =0.267	β = 1.174 [0.255; 2.093] *p*-adj = 0.242
Tri	0.057	90.372 [67.77; 112.98]	β = −19.442 [−64.65; 25.77] *p*-adj = 0.867	β = 0.224 [−1.46; 1.91] *p*-adj = 0.800	β = −0.860 [−4.22; 2.5] *p*-adj = 0.886
HDL	0.008	65.296 [55.105; 75.49]	β = 2.339 [−18.04; 22.72] *p*-adj = 0.999	β = 0.297 [−0.488; 1.08] *p*-adj = 0.485	β = 0.211 [−1.36; 1.78] *p*-adj = 0.886
TC		189.961 [173.91; 206]	β = 0.501 [−31.60; 32.599] *p*-adj = 0.999	β = −2.372 [−4.88; 0.133] *p*-adj = 0.267	β = −0.970 [−5.98; 4.040] *p*-adj = 0.886
LDL	0.0877	100.179 [89.86; 110.496]	β = −0.001 [−20.65; 20.61] *p*-adj = 0.999	β = −1.565 [−3.12; −0.007] *p*-adj = 0.267	β = −0.404 [−3.52; 2.71] *p*-adj = 0.886
Apo A1/B	0.028	0.525 [0.445; 0.606]	β = 0.010 [−0.150; 0.171] *p*-adj = 0.999	β = −0.004 [−0.009; 1.59 × 10^−4^] *p*-adj = 0.267	Β = −0.009 [−0.019; 7.65 × 10^−4^] *p*-adj = 0.611
eGFR	0.060	98.676 [89.535; 107.816]	β= 7.699 [−10.582; 25.98] *p*-adj = 0.867	β = −0.211 [−0.57; 0.148] *p*-adj = 0.445	β= −0.381 [−0.338; 1.099] *p*-adj = 0.886
ESR	0.155	14.527 [9.957; 19.097]	β= 7.230 [−1.910; 16.37] *p*-adj = 0.867,	β= −0.185 [−0.810; 0.440] *p*-adj = 0.641	β= −0.322 [−1.571; 0.928] *p*-adj = 0.886

**Table 4:** Linear Mixed-Effects Model analysis evaluating the effects of the IHHT protocol, training sessions, and their interaction on oxidative stress and inflammatory biomarkers. Data are presented as β estimates with 95% Confidence Intervals [Lower; Upper] and FDR-adjusted *p*-values (*p*-adj). Intercept: Baseline reference value for the Training Group (TG). Conditioning: Main effect of the assigned IHHT protocol. The β estimate reflects the mean difference in the Conditioning Group (CG) compared to the Training Group (TG). Training: Main effect of time (number of sessions). The β estimate represents the average change in the biomarker per training session. C × T (Interaction): Time by conditioning interaction. The β estimate indicates how the change over time in the CG differs from the change observed in the TG. Abbreviations: ROS (Reactive Oxygen Species, [ μmol/min]); TAC (Total Antioxidant Capacity, [mM]); 8-ISO (8-isoprostane, [ng/mg]); IL-6 (Interleukin 6, [pg/mL]); 3NT (3-Nitrotyrosine, [nM/L]); NOX (Nitric Oxide Total Metabolites, [μM]); NO_2_ (Nitrite, [μM]). *p*-adj: indicates adjusted *p*-values calculated through the Benjamini–Hochberg False Discovery Rate method to correct for multiple comparisons.

Parameter	R-Squared-Marginal	Intercept	Conditioning β (Estimate) *p*-Values 95% CI [L, U]	Training β (Estimate) *p*-Values 95% CI [L, U]	C × T β (Estimate) *p*-Values 95% CI [L, U]
ROS	0.528	1.054 [0.96; 1.14]	β= −0.094 [−2.7; 0,08] *p*-adj = 0.517	β= 0.12 [0.09; 0.15] *p*-adj = < 0.001	β= 0.009 [−0.05; 0.07] *p*-adj = 0.756
TAC	0.245	0.919 [0.69; 1.14]	β= −0.02 [−0.4; 0.4] *p*-adj = 0.900	β= −0.099 [−0.1; −0.06] *p*-adj = < 0.001	β= 0.04 [−0.02; 0.12] *p*-adj = 0.376
8-ISO	0.473	437.3 [349.5; 525.06]	β= −161 [−354.6; 3.45] *p*-adj = 0.333	β= 68.7 [−49.7; 87.6] *p*-adj = < 0.001	β= −28.5 [−66.5; 9.37] *p*-adj = 0.376
IL-6	0.473	6.604 [5.20; 7.99]	β= −2.621 [−5.41; 0.17] *p*-adj = 0.333	β= 0.978 [0.67; 1.27] *p*-adj = < 0.001	β= −0.961 [−1.55; −0.36] *p*-adj = 0.021
3NT	0.057	56.21 [50.49; 61.92]	β= −5.49 [−16.93; 5.94] *p*-adj = 0.517	β= −0.55 [−1.44; 0.32] *p*-adj = 0.220	β= −0.659 [−2.42; 1.09] *p*-adj = 0.544
NOX	0.075	1194 [638; 1749.6]	β= 386.8 [−724; 1498] *p*-adj = 0.595	β= −99.2 [−172; −26.2] *p*-adj = 0.015	β= −63.1 [−209; 82.9] *p*-adj = 0.544
NO_2_	0.163	1.387 [0.65; 2.12]	β= −1.15 [−2.6; 0.3] *p*-adj = 0.359	β= 0.137 [0.01; 0.25] *p*-adj = 0.034	β= −0.159 [−0.39; 0.07] *p*-adj = 0.376

**Table 5:** Linear Mixed-Effects Model analysis evaluating the effects of the IHHT protocol, training sessions, and their interaction on renal function and immune response parameters. Data are presented as β estimates with 95% Confidence Intervals [Lower; Upper] and FDR-adjusted *p*-values (*p*-adj). Intercept: Baseline reference value for the Training Group (TG). Conditioning: Main effect of the assigned IHHT protocol. The β estimate reflects the mean difference in the CG compared to the TG. Training: Main effect of time (number of sessions). The β estimate represents the average change in the biomarker per training session. C × T (Interaction): Time by conditioning interaction. The β estimate indicates how the change over time in the CG differs from the change observed in the TG. Abbreviations: creatinine ([g/L]); neopterin ([nM/mol]); uric acid ([mg/dL]). *p*-adj indicates the adjusted *p*-values calculated using the Benjamini–Hochberg False Discovery Rate method to correct for multiple comparisons.

Parameter	R-Squared-Marginal	Intercept	Conditioning β (Estimate) *p*-Values 95% CI [L, U]	Training β (Estimate) *p*-Values 95% CI [L, U]	T × C β (Estimate) *p*-Values 95% CI [L, U]
C	0.193	0.973 [0.77; 1.17]	β= −0.16 [−0.56; 0.24] *p*-adj = 0.513	β= 0.07 [0.03; 0.11] *p*-adj = < 0.001	β= −0.05 [−0.13; −0.023] *p*-adj = 0.309
Neopterin	0.242	178.8 [129.3; 228.23]	β= −34.2 [−133.1; 64.64] *p*-adj = 0.513	β= 19.2 [12.1; 26.43] *p*-adj = < 0.001	β= −12.2 [−26.6; 2.17] *p*-adj = 0.309
Uric Acid	0.079	20.94 [15.65; 26.25]	β= −7.9 [−18.51; 2.69] *p*-adj = 0.513	β= 0.03 [−1.09; 1.02] *p*-adj = 0.949	β= −0.52 [−2.65; 1.59] *p*-adj = 0.627

## Data Availability

The data that support the findings of this study are available from the corresponding authors, Manuel Marzola and Tommaso Antonio Giacon, upon reasonable request.

## Abbreviations

The following abbreviations are used in this manuscript:

AMS	Acute Mountain Sickness
CG	Conditioning Group
CMH	1-Hydroxy-3-Methoxy-Carbonyl-2,2,5,5-Tetramethylpyrrolidine
CP	3-Carboxy-2,2,5,5-Tetramethyl-1-Pyrrolidinyloxy
ELISA	Enzyme-Linked Immunosorbent Assay
EPR	Electron Paramagnetic Resonance
ERS	Erythrocyte Sedimentation Rate
eGFR	Estimated Glomerular Filtration rate
HDL	High-Density Lipoprotein
HIF-1α	Hypoxia-Inducible Factor 1α
HPLC	High-Performance Liquid Chromatography
HR	Heart Rate
IHHT	Intermittent Hypoxic–Hyperoxic Training
IL-6	Interleukin-6
IL-10	Interleukin-10
LDL	Low-Density Lipoprotein
LL	Lake Louise Score
LLTH	Live Low–Train High
METs	Metabolic Equivalents
NO_2_	Nitrite
NO_3_	Nitrate
NO_x_	Nitric Oxide Metabolites
Nrf2	Transcription Factor Nrf2
ROS	Reactive Oxygen Species
SpO_2_	Peripheral Oxygen Saturation
TAC	Total Antioxidant Capacity
TG	Training Group
3-NT	3-Nitrotyrosine
8-iso-PGF2α	8-Isoprostane-ProstaglandinF-2alfa

## Appendix A

**Table A1:** Biochemical panel parameters measured before (Pre) and after (Post) the IHHT protocol. Data are presented as mean ± SD (standard deviation).

	Pre IHHT		Post IHHT	
Parameters	Mean	SD	Mean	SD
reticulocytes	0.838	0.234	0.965	0.252
absolute fractions	40.345	11.38	46.81	15.385
immature fractions	35.9	10.38	34	7.77
glucose	88.4	5.37	83.1	3.84
triglycerides	90.9	38.9	91.7	42.58
HDL	64.08	14.71	66.9	21.53
LDL	105.75	21.62	92.1	15.98
total cholesterol	198.25	33.24	178.7	26.52
Apo A1/B	0.52	0.12	0.5	0.15
creatinine	0.91	0.15	0.93	0.11
eGFR	98.9	16.37	97	13.4
ESR	14.5	9.8	12	6.7
